# Discrepancy in SARS-CoV-2 Infection Status Among PCR, Serological, and Cellular Immunity Assays of Nucleocapsids: A Historical Cohort Study

**DOI:** 10.3390/vaccines13030259

**Published:** 2025-02-28

**Authors:** Taiga Uchiyama, Yurie Kobashi, Takeshi Kawamura, Yoshitaka Nishikawa, Aya Nakayama, Fumiya Oguro, Yudai Kaneko, Chika Yamamoto, Naomi Ito, Tianchen Zhao, Hiroaki Saito, Toshiki Abe, Tatsuhiko Kodama, Masaharu Tsubokura

**Affiliations:** 1 Department of Radiation Health Management, Fukushima Medical University School of Medicine, Fukushima City 960-1295, Fukushima, Japan; u0807892@gmail.com (T.U.); tsubo-m@fmu.ac.jp (M.T.); 2 Global Exchange Center, Fukushima Medical University School of Medicine, Fukushima City 960-1247, Fukushima, Japan; 3 Department of General Internal Medicine, Hirata Central Hospital, Hirata 963-8202, Fukushima, Japan; 4 Proteomics Laboratory, Isotope Science Center, The University of Tokyo, Tokyo 113-8654, Japan; 5 Laboratory for Systems Biology and Medicine, Research Center for Advanced Science and Technology, The University of Tokyo, Tokyo 113-8654, Japan; 6 Medical & Biological Laboratories Co., Ltd., Tokyo 105-0012, Japan; 7 General Incorporated Association for Comprehensive Disaster Health Management Research Institute, Tokyo 108-0074, Japan

**Keywords:** COVID-19, SARS-CoV-2, humoral immunity, cellular immunity, nucleocapsid

## Abstract

**Background/Objectives**: Limited research has compared tests assessing humoral and cellular immunity related to SARS-CoV-2 infection. This study evaluated immunoglobulin G for nucleocapsid (IgG(N)) and T-spot for nucleocapsid (T-spot(N)) assays against polymerase chain reaction (PCR) test results for identifying infected individuals. **Methods**: This study included participants who had completed five blood samplings since their second COVID-19 vaccination between 9 September 2021 and 6 November 2022. Chemiluminescent immunoassay (CLIA) tests measured the humoral immune response, IgG(S) and neutralizing activity tests the immune status, and IgG(N) tests the infection history. For cellar immunity, T-spot(S) indicated immune status, and T-spot(N) indicated infection history. **Results**: The primary outcome was the proportion of individuals who tested positive for PCR and the proportion who tested positive for IgG(N) and T-spot(N). Overall, this study included 2104 participants. In the PCR-negative group, 1838 individuals tested negative for IgG(N), whereas 64 tested positive at least once. The geometric mean of IgG(S) at T5 was 1541.7 AU/mL in the IgG(N)-negative group and 3965.8 AU/mL in the IgG(N)-positive group, which was 2.6 times higher. In the PCR-positive group, 25 individuals tested negative for IgG(N), while 177 tested positive at least once. The geometric mean of IgG(S) at T5 was 2700.6 AU/mL in the IgG(N)-negative group and 5400.8 AU/mL in the IgG(N)-positive group, showing higher values in the IgG(N)-positive group. **Conclusions**: A discrepancy was noted between PCR test results and the IgG(N) and T-spot(N) determinations. Combining multiple assays is required to accurately identify the past-infected population.

## 1. Introduction

Since its initial detection in China in December 2019, severe acute respiratory syndrome coronavirus 2 (SARS-CoV-2) has rapidly spread worldwide, causing a global pandemic. This highly contagious virus spreads primarily through droplet transmission, contact transmission, and aerosol transmission, with droplets being the main mode of propagation [1]. Effective infection control measures include isolating infected individuals and preventing contact between infected and non-infected individuals. In clinical settings, the SARS-CoV-2 diagnosis relies on polymerase chain reaction (PCR), antigen, and antibody tests. PCR and antigen tests primarily detect current infections, while antibody tests can indicate past or ongoing infections, particularly in later stages. PCR tests offer high accuracy yet require specialized equipment and skilled operators, making them costly and time-consuming. Rapid antigen tests typically yield quick results in 15 to 30 min and are simple and inexpensive [2]. However, their sensitivity is lower than that of PCR tests, often resulting in false-negative outcomes [3]. Addressing these limitations in detecting SARS-CoV-2 infection is crucial for swiftly and accurately identifying infected individuals to prevent further spread.

Numerous studies have explored strategies to efficiently identify infected individuals by combining various tests, each with its own advantages and disadvantages. Each test often presents false positives or false negatives. For example, RT-PCR tests may yield negative results during the initial days of infection or after recovery, when the viral load is low. Antibody tests may also be negative early in the infection, owing to the absence of antibodies, whereas, in previously infected individuals, cross-reactivity might cause positive results despite the lack of current infection. Antigen tests targeting specific and highly reactive viral antigens that precede antibody production are less likely to present false positives or negatives [4]. Additionally, a portion of SARS-CoV-2-infected individuals are asymptomatic [5]. Asymptomatic carriers of COVID-19 can transmit SARS-CoV-2, with their infectivity being similar to that of symptomatic patients [6]. Despite such studies, few have compared multiple tests to evaluate both humoral and cellular immunity concerning SARS-CoV-2 infection.

On 8 May 2023, Japan eased regulations on SARS-CoV-2, prompting the Ministry of Health, Labour and Welfare to shift from comprehensive monitoring to sentinel surveillance to understand the epidemic status of COVID-19. This change made tracking COVID-19 cases difficult, leading to outbreaks in medical facilities and schools, highlighting its significance in Japan. Fukushima Prefecture, located in the Tohoku region, experienced unprecedented devastation from the 2011 earthquake in Great East Japan and the Fukushima Daiichi Nuclear Power Plant accident [7]. These experiences fostered ongoing collaboration across community, local government, and public and private healthcare sectors for over a decade [8,9,10]. During the COVID-19 pandemic, leveraging this regional cooperation, the Fukushima Vaccine Community Survey was conducted in Hirata Village, Soma City, and Minamisoma City within Fukushima Prefecture [11,12]. This survey involved regular blood sampling of residents and healthcare workers to observe changes in SARS-CoV-2 antibody titers post-vaccination. Approximately 2500 individuals participated, making it one of the most comprehensive sources of information on post-vaccination antibody titers nationwide. Hence, this region is ideal for community-level evaluation of various antibody titers and test results.

Various testing methods are employed to diagnose COVID-19; however, few studies have compared the results from multiple methods. This study aimed to compare the results of multiple tests conducted within the same cohort to determine SARS-CoV-2 infection during the pandemic. IgG(N) and T-spot(N) determinations were compared with PCR results to identify infected individuals.

## 2. Materials and Methods

### 2.1. Study Design and Participants

**Figure 1:**
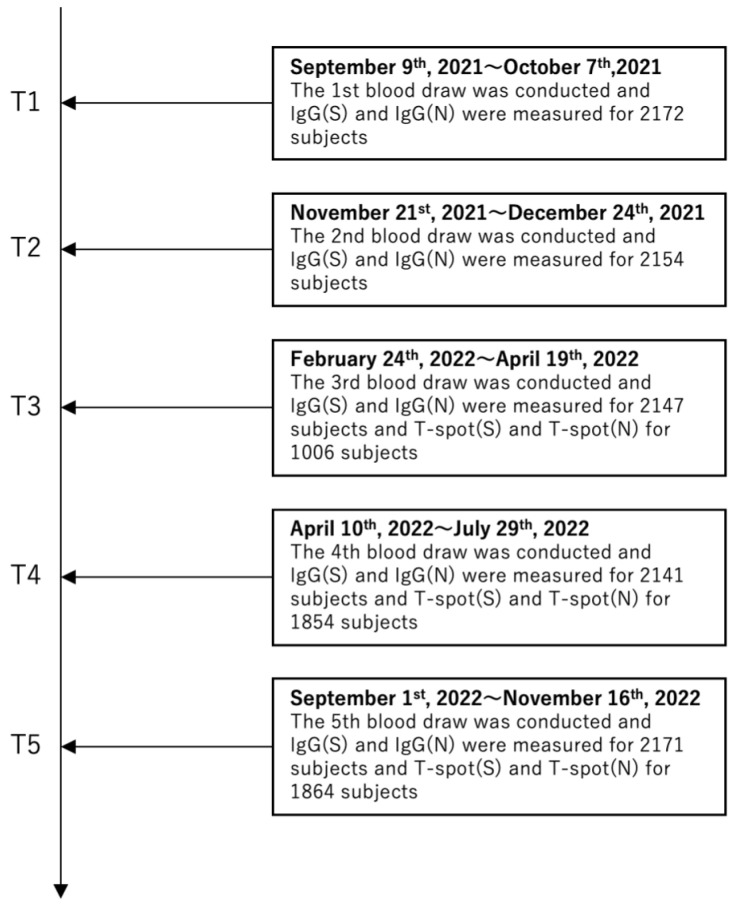
The timeline for the five blood samplings.

This study is an observational historical cohort study that selected participants from the Fukushima cohort study for the Fukushima Vaccination Cohort Survey. Blood sampling occurred as follows: the first from 9 September to 7 October 2021 (T1); the second from 21 November to 24 December 2021 (T2); the third from 24 February to 19 April 2022 (T3); the fourth from 10 April to 29 July 2022 (T4); and the fifth from 1 September to 6 November 2022 (T5) (Figure 1). The blood sampling method was described elsewhere [11]. In total, 2527 participants were included in the entire cohort, and 354 participants were excluded without the infection status data. The study focused on individuals who completed all five samplings after their second COVID-19 vaccination; sixty-nine participants who did not have all of the data on five blood tests were excluded. Overall, 2104 participants were included in the final analysis. Data on participants’ age, sex, type of vaccine received, smoking history, drinking history, medication history, comorbidities, symptoms during infection, and consent to participate in the study were collected via a questionnaire survey. Regarding alcohol consumption, individuals who have opportunities to drink in their daily lives, including occasional or social drinking, were classified as alcohol consumers. At each sampling visit, a questionnaire was also used to investigate whether participants had undergone a PCR test since the last visit. If so, the PCR results (positive/negative) and the date of the PCR test were recorded. PCR tests were performed on healthcare providers and patients who had subjective symptoms such as fever or when clusters occurred at the healthcare facility. Written informed consent was obtained from all participants. This study was approved by the ethics committees of Hirata Central Hospital (number 2021-0611-1) and Fukushima Medical University (number 2021-116).

### 2.2. Serological Testing

Blood samples were processed by centrifugation to obtain serum-only samples, which were subsequently sent to the University of Tokyo for analysis. IgG antibody titers against the SARS-CoV-2 spike (S) protein, IgG antibody titers against the SARS-CoV-2 nucleocapsid (N) protein, and neutralizing activity were measured using chemiluminescent immunoassays with iFlash 3000 (YHLO Biotech, Shenzhen, China) and iFlash-2019-nCoV series reagents (YHLO Biotech). IgG(S) and neutralizing activity were used as indicators of immune status, whereas IgG(N) served as an indicator of infection history. The cut-off values of IgG(S), IgG(N), and neutralizing activity were 10 AU/mL.

### 2.3. Cellular Immune Response Measurements

Cellular immune responses were evaluated using the T-Spot COVID test (Oxford Immunotec, Abingdon, Cambridge, UK), an ELISpot interferon-γ release assay. Peripheral blood samples (10 mL) were collected in heparin lithium and EDTA tubes. Following the addition of T-cell Xtend reagent (Oxford Immunotec) and density gradient centrifugation to isolate peripheral blood mononuclear cells (PBMCs), 250,000 PBMCs per participant were added to each of the four wells: negative control, positive control, SARS-CoV-2 spike antigen, and SARS-CoV-2 nucleocapsid antigen. Stimulation of T cells in the SARS-CoV-2 antigen wells induced INF-γ production, detected by AP-labeled secondary antibodies and substrates to form INF-γ-producing spots. The results were interpreted by subtracting the number of spots in the negative control well from the number of spots in each well. Results interpretation followed official guidelines: <5 spots per well indicated non-reactivity, 5 to 7 spots was inconclusive, and ≥8 spots indicated reactivity. Tests with >10 spots in the negative control well were deemed invalid.

### 2.4. Primary Outcome

The primary outcome was the proportion of individuals who tested positive for PCR, IgG(N), and T-spot(N).

### 2.5. Statistical Analyses

First, descriptive summaries of participant information were compiled. Positive results for IgG(N) and T-spot(N) were descriptively summarized for individuals with and without infection. Geometric mean values of IgG(S) at T5 were presented for individuals who tested positive for IgG(N) at least once across all tests and those who never tested positive, categorized by infection history. Similarly, geometric mean values of T-spot(S) at T5 were shown for each group. Next, the relationships between different assays were figured. Then, symptoms among infected participants within each group of IgG(N), negative or positive, were summarized. Continuous or categorical variables are shown as median (interquartile) or number (percentage). The analysis in this study is descriptive, with categorical variables expressed as frequencies and percentages and antibody titers as geometric means and 95% confidence intervals. All statistical analyses were performed using STATA/IC15.

## 3. Results

### 3.1. Participant Characteristics

The number of participants for each blood test (T1–T5) is shown in Figure 1. The serological assay was performed between the T1 and T5 blood samplings; however, the cellular immune responses assay was performed only between the T3 and T5 blood samplings. Table 1 summarizes the demographic and health characteristics of the 2373 study participants. The average age of participants was 52 [38–68] years. The cohort comprised 1220 women and 884 men, with women accounting for 58.0% of the participants. By the fifth blood draw, 2070 individuals had received up to the third vaccine dose, and 1406 had received up to the fourth dose.

### 3.2. Relationship Between Infection Status and IgG(N) and T-spot(N)

Table 2 displays the frequency of positive results for IgG(N) and T-spot(N). Among the 1902 non-infected individuals, 40 (2.1%) were positive once for IgG(N), 14 (0.7%) twice, 1 (0.1%) three times, and 9 (0.5%) five times. Among the 202 infected individuals, 24 (11.9%) consistently tested negative for IgG(N). For T-spot(N), among the 865 non-infected individuals, 45 (5.2%) tested positive once and 60 (6.5%) twice. Among the 115 infected individuals, 48 (41.7%) tested negative for T-spot(N). The number of participants per frequency of positive IgG(N) and T-spot(N) among those who had all five IgG tests and all three T-spot tests were showed in Table S1.

### 3.3. Grouping by Infection History and IgG(N) Determination

For IgG(S), among the non-infected group, 1838 were IgG(N)-negative, and 64 were IgG(N)-positive at least once (Table 3). For non-infected participants, the geometric mean of IgG(S) at T5 was 1541.7 AU/mL in the IgG(N)-negative group and 3965.8 AU/mL in the IgG(N)-positive group, the latter being 2.57 times higher. Similarly, among the infected group, 25 were IgG(N)-negative, and 177 were IgG(N)-positive at least once. The geometric mean of IgG(S) at T5 was 2700.6 AU/mL in the IgG(N)-negative group and 5400.8 AU/mL in the IgG(N)-positive group, the latter being 2.0 times higher.

For T-spot(S), among the non-infected group, 1525 were IgG(N)-negative, and 52 were IgG(N)-positive at least once (Table 3). The geometric mean of T-spot(S) at T5 was 12.6 spots in the IgG(N)-negative group and 15.2 spots in the IgG(N)-positive group. Similarly, among the infected group, 17 were IgG(N)-negative, and 161 were IgG(N)-positive at least once. The geometric mean of T-spot(S) at T5 was 17.9 spots in the IgG(N)-negative group and 21.2 spots in the IgG(N)-positive group, the latter being 1.2 times higher.

### 3.4. Grouping by Infection History and T-spot(N) Status

For IgG(S), among the non-infected group, 789 were T-spot(N)-negative, and 72 were T-spot(N)-positive at least once (Table 4). For non-infected participants, the geometric mean of IgG(S) at T5 was 1556.7 AU/mL in the T-spot(N)-negative group and 2268.2 AU/mL in the T-spot(N)-positive group, the latter being 1.5 times higher. Similarly, among the infected group, 48 were T-spot(N) negative, and 70 were T-spot(N)-positive at least once. The geometric mean of IgG(S) at T5 was 4872.7 AU/mL in the T-spot(N)-negative group and 5277.0 AU/mL in the T-spot(N)-positive group, the latter being 1.1 times higher.

For T-spot(S), among the non-infected group, 755 were T-spot(N)-negative, and 71 were T-spot(N)-positive at least once (Table 4). For non-infected participants, the geometric mean of T-spot(S) at T5 was 11.4 spots in the T-spot(N)-negative group and 15.0 spots in the T-spot(N)-positive group, the latter being 1.3 times higher. Similarly, among the infected group, 48 were T-spot(N) negative, and 70 were T-spot(N)-positive at least once. For infected participants, the geometric mean of T-spot(S) at T5 was 15.1 spots in the T-spot(N)-negative group and 26.4 spots in the T-spot(N)-positive group, the latter being 1.8 times higher.

### 3.5. Relationships Between Different Assays

**Figure 2:**
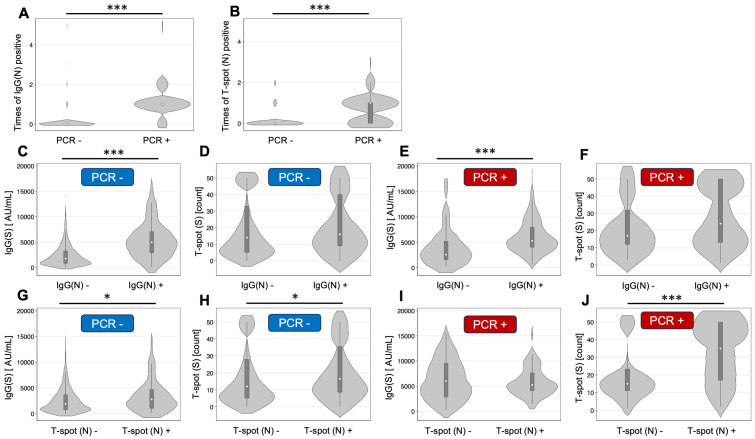
Relationships between different assays. (**A**) IgG(N)-positive times by the results of PCR. (**B**) T-spot (N)-positive times by the results of PCR. (**C**) IgG(S) titer at T5 by the group of IgG(N) results and negative PCR. (**D**) T-spot(S) at T5 by the group of IgG(N) results and negative PCR. (**E**) IgG(S) titer at T5 by the group of IgG(N) results and positive PCR. (**F**) T-spot(S) at T5 by the group of IgG(N) results and positive PCR. (**G**) IgG(S) titer at T5 by the group of T-spot (N) results and negative PCR. (**H**) T-spot(S) at T5 by the group of T-spot (N) results and negative PCR. (**I**) IgG(S) titer at T5 by the group of T-spot (N) results and positive PCR. (**J**) T-spot(S) at T5 by the group of T-spot (N) results and positive PCR. * *p* < 0.05, *** *p* < 0.001 with Wilcoxon rank sum test.

A summary of the relationships between different assays is shown in Figure 2. There were significant differences in the times of IgG(N) positivity by the results of PCR and the times of T-spot (N) positivity by the results of PCR (*p* < 0.001). The IgG(S) titers at T5 by the group of IgG(N) results were significantly different in both the positive PCR group and the negative PCR group (*p* < 0.001).

### 3.6. Symptoms Among Infected Participants

Symptoms among the infected participants within each group, IgG(N)-negative or -positive, were descriptively analyzed. Only sore throat was significantly higher among IgG(N)-positive groups (Table 5, *p* = 0.042).

## 4. Discussion

Accurate diagnosis of COVID-19 infected status among the population was important to the design of the strategy regarding infection control. In this study, five blood samples were collected from rural Japanese healthcare workers and residents between September 2021 and November 2022. IgG(N) and T-spot(N) results were compared with PCR findings to determine COVID-19 infection.

A discrepancy was noted between the PCR results and IgG(N) and T-spot(N) measurements. In this study, 2.1% (40/1902) and 8.5% (45/865) of participants tested positive for IgG(N) and T-spot(N), respectively, despite negative PCR results, indicating previous infection. The geometric mean values of IgG(S) and T-spot(S) at T5 were 1541.7 AU/mL and 12.6 in the IgG(N)-negative group and 3965.8 AU/mL and 15.2 in the IgG(N)-positive group, with geometric mean ratios of 2.6 and 1.2, respectively. These individuals likely had low viral loads or asymptomatic infection at the time of the study. Prior studies have shown that early infection and periods of convalescence often result in negative PCR tests due to low viral loads [3,13,14]. Additionally, some people have subclinical infections consistent with these results [15,16,17,18]. Multiple tests may be necessary to accurately determine true infection status [19,20,21,22].

Some individuals infected with coronaviruses had a weak immune response. In this study, 12.3% (25/202) and 41.7% (48/115) of participants had negative IgG(N) and T-spot(N), respectively, despite positive PCR tests. The geometric mean values of IgG(S) and T-spot(S) at T5 were 2700.6 AU/mL and 17.9 in the IgG(N)-negative population and 5400.8 AU/mL and 21.2 in the IgG(N)-positive population, with geometric mean ratios of 2.0 and 1.2, respectively. This means that IgG(S) antibody titers in PCR-positive, IgG(N)-negative individuals were 50.0% lower than those in IgG(N)-positive individuals. In a previous study, IgG(S) antibody titers were higher in IgG(N)-positive than in IgG(N)-negative individuals without a history of previous disease, consistent with these results [23]. These populations may include numerous people with inadequate IgG(N) and IgG(S) production, i.e., a reduced ability to produce antibodies. For example, people with underlying diseases, such as older patients or those undergoing hemodialysis, have low antibody titers after vaccination [23,24,25]. Given that some populations have reduced humoral and cellular immunity, using methods that do not rely on antibody production, such as PCR and antigen testing, is crucial to determine the infection history of these individuals.

SARS-CoV-2 infection may not be simply divided into infected or uninfected. Participants were classified into four groups based on PCR and IgG(N) test results. The IgG(S) antibody titers at T5 were PCR-positive and IgG(N)-positive (5400.8 AU/mL), PCR-positive and IgG(N)-negative (2700.6 AU/mL), PCR-negative and IgG(N)-positive (3965.8 AU/mL), and PCR-negative and IgG(N)-negative (1541.7 AU/mL). In summary, even with the same PCR results, antibody titers varied based on IgG(N) results, indicating that PCR alone may not fully categorize infection status. New models are needed for a comprehensive understanding of infectivity.

This study had several limitations. First, the study was biased toward a predominantly female population, with most of the participants being healthcare professionals (57.4% female). Second, the chemiluminescence immunoassay and T-Spot COVID-19 test used were research methods whose applicability in clinical settings is unclear. Third, the tests were conducted during the prevalence of the BA-5 strain, which limited their generalizability to other strains over time. Fourth, we did not clarify the period of PCR results performed before the first blood sampling. Fifth, we did not measure the viral load, and the assessment and interpretation of the time interval between the PCR test and antibody test were difficult; thus, we did not include this information. Despite these limitations, this is the first study to classify participants into several groups based on IgG(N) and T-spot(N) determinations and PCR results and to compare antibody titers and cellular immune response measurements for each group.

## 5. Conclusions

A discrepancy was noted between PCR test results and the IgG(N) and T-spot(N) determinations. Classifying individuals definitively as infected or non-infected based on a single test, such as PCR, may be challenging. Therefore, combining several types of assays is necessary to accurately identify the infected population. Additionally, differences in antibody titers were observed based on IgG(N) results, even among individuals with the same PCR outcomes. A comprehensive classification and method are required to understand infection status in detail.

## Figures and Tables

**Table 1:** Characteristics of the participants in this study (n = 2104).

Variable	n (%)
Age	52 (39–67)
Under 41	572 (27.2)
41–65	977 (46.4)
Over 65	555 (26.3)
Sex: female	1220 (58.0)
Type of third dose vaccination (n = 2070)	
mRNA-1273	1401 (67.7)
BNT162b	669 (32.3)
Type of fourth dose vaccination (n = 1406)	
mRNA-1273	337 (24.0)
BNT162b	1008 (71.7)
Others	61 (4.3)
Smoking habit (n = 2069)	392 (19.0)
Alcohol consumption (n = 2058)	904 (43.9)
Daily medicine	
Steroid (n = 2072)	40 (1.9)
Immunosuppression (n = 2067)	21 (1.0)
Biologics (n = 2065)	11 (0.5)
Comorbidity	
Hypertension (n = 2098)	555 (26.5)
Diabetes (n = 2098)	155 (7.4)
Dyslipidemia (n = 2098)	248 (11.8)
BMI (n = 1923)	
Under 18.5	120 (6.2)
18.5–under 25	1221 (63.5)
25–30	443 (23.0)
Over 30	139 (7.2)

**Table 2:** Number of persons per number of times the IgG(N) and T-spot(N) were positive (n = 2104 for IgG(N), n = 980 for T-spot(N)).

	Not Infected	Infected	Infected and Hospitalized
IgG(N)			
Never	1838	24	1
1 time	40	142	3
2 times	14	26	4
3 times	1	0	0
4 times	0	0	0
5 times	9	2	0
T-spot(N)			
Never	789	45	3
1 time	50	60	0
2 times	22	9	1
3 times	0	1	0

**Table 3:** Geometric mean (95% CI) of IgG(S) and T-spot(S) at T5 for each infection status group and IgG(N)-positive status group.

	Geometric Mean (95% CI)
**IgG(S) at T5**	
**Not infected (with PCR test)**	
IgG(N)-negative (n = 1838)	1541.7 (1469.8–1617.0)
IgG(N)-positive between T1 and T5 (n = 64)	3965.8 (3071.0–5121.4)
**Infected (with PCR test)**	
IgG(N)-negative (n = 25)	2700.6 (1800.6–4050.5)
IgG(N)-positive between T1 and T5 (n = 177)	5400.8 (4963.4–5876.8)
**T-spot(S) at T5**	
**Not infected (with PCR test)**	
IgG(N)-negative (n = 1525)	12.6 (11.9–13.3)
IgG(N)-positive between T1 and T5 (n = 52)	15.2 (11.2–20.6)
**Infected (with PCR test)**	
IgG(N)-negative (n = 17)	17.9 (11.8–27.1)
IgG(N)-positive between T1 and T5 (n = 161)	21.2 (18.6–24.3)

**Table 4:** Geometric mean (95% CI) of IgG(S) and T-spot(S) at T5 for each infection status group and T-spot(N)-positive status group.

	Geometric Mean (95% CI)
**IgG(S) at 5T**	
**Not infected (with PCR test)**	
T-spot(N)-negative (n = 789)	1556.7 (1435.1–1688.6)
T-spot(N)-positive between T1 and T5 (n = 72)	2268.2 (1770.3–2906.1)
**Infected (with PCR test)**	
T-spot(N)-negative (n = 48)	4872.7 (3824.6–6208.1)
T-spot(N)-positive between T1 and T5 (n = 70)	5277.0 (4668.8–5964.3)
**T-spot(S) at 5T**	
**Not infected (with PCR test)**	
T-spot(N)-negative (n = 755)	11.4 (10.5–12.3)
T-spot(N)-positive between T1 and T5 (n = 71)	15.0 (11.6–19.1)
**Infected (with PCR test)**	
T-spot(N)-negative (n = 48)	15.1 (11.8–19.4)
T-spot(N)-positive between T1 and T5 (n = 70)	26.4 (21.7–32.0)

**Table 5:** Symptoms among infected participants within each group of IgG(N)-negative or -positive (n = 202).

	IgG(N)-Negative (n = 25)	IgG(N)-Positive (n = 177)	*p*-Value
Fever	19 (79.2)	131 (74.0)	0.59
Dysgeusia	2 (8.3)	33 (18.6)	0.21
Cough	13 (54.2)	121 (68.4)	0.166
Sore throat	10 (41.7)	112 (63.3)	0.042
Headache	9 (37.5)	74 (41.8)	0.69
Joint and muscle pain	6 (25.0)	51 (28.8)	0.70
Diarrhea	2 (8.3)	27 (15.3)	0.37
Rash	0 (0.0)	4 (2.3)	0.46
Fatigue	10 (41.7)	107 (60.5)	0.080
Eye symptoms	0 (0.0)	2 (1.1)	0.60
Difficulty breathing	2 (8.3)	33 (18.6)	0.21
Chest pain	1 (4.2)	8 (4.5)	0.94
Difficulty talking	1 (4.2)	4 (2.3)	0.58

## Data Availability

The datasets generated for this study are not available publicly; however, they can be accessed upon request to the corresponding author with the permission of Fukushima Medical University and Hirata Central Hospital.

## Supplementary Materials

The following supporting information can be downloaded at https://www.mdpi.com/article/10.3390/vaccines13030259/s1, Table S1: Number of participants per frequency of positive IgG(N) and T-spot(N) for nucleocapsids among those who had all five IgG tests and all three T-spot tests (n = 983).

## Abbreviations

The following abbreviations are used in this manuscript:

IgG(N)	Immunoglobin G for nucleocapsid
PCR	Polymerase chain reaction
SARS-CoV-2	Severe acute respiratory syndrome coronavirus 2
CLIA	Chemiluminescent immunoassay
