# Supplementary material for: Discrepancy in SARS-CoV-2 Infection Status Among PCR, Serological, and Cellular Immunity Assays of Nucleocapsids: A Historical Cohort Study
Source: Vaccines (Basel). 2025 Feb 28;13(3):259. doi: 10.3390/vaccines13030259 (PMC11945907; doi:10.3390/vaccines13030259)
Supplement: Supplementary file 1 [file vaccines-13-00259-s001.zip › vaccines-3432452-supplementary.pdf]

SUPPLEMENTAL TABLE 1 Number of participants per frequency of positive IgG(N) and T-spot(N) for nucleocapsids among those who had all five IgG tests and all three T-spot tests (n = 983).

	T-spot (N)				
	Never	1 time	2 times	3 times	Total
IgG(N)					
Never	781	48	20	0	849
1 time	41	57	5	0	103
2 times	12	5	9	1	27
3 times	0	0	0	0	0
4 times	0	0	0	0	0
5 times	4	0	0	0	4
Total	838	110	34	1	983
